# 3D‐Printed Strong Dental Crown with Multi‐Scale Ordered Architecture, High‐Precision, and Bioactivity

**DOI:** 10.1002/advs.202104001

**Published:** 2021-12-22

**Authors:** Menglu Zhao, Danlei Yang, Suna Fan, Xiang Yao, Jiexin Wang, Meifang Zhu, Yaopeng Zhang

**Affiliations:** ^1^ State Key Laboratory for Modification of Chemical Fibers and Polymer Materials Shanghai Belt and Road Joint Laboratory of Advanced Fiber and Low‐dimension Materials College of Materials Science and Engineering Donghua University Shanghai 201620 P. R. China; ^2^ State Key Laboratory of Organic‐Inorganic Composites Beijing University of Chemical Technology Beijing 100029 P. R. China

**Keywords:** 3D printing, finite element method, hierarchical architecture, hydroxyapatite, mechanical properties

## Abstract

Mimicking the multi‐scale highly ordered hydroxyapatite (HAp) nanocrystal structure of the natural tooth enamel remains a great challenge. Herein, a bottom‐up step‐by‐step strategy is developed using extrusion‐based 3D printing technology to achieve a high‐precision dental crown with multi‐scale highly ordered HAp structure. In this study, hybrid resin‐based composites (RBCs) with “supergravity +” HAp nanorods can be printed smoothly via direct ink writing (DIW) 3D printing, induced by shear force through a custom‐built nozzle with a gradually shrinking channel. The theoretical simulation results of finite element method are consistent with the experimental results. The HAp nanorods are first highly oriented along a programmable printing direction in a single printed fiber, then arranged in a layer by adjusting the printing path, and finally 3D printed into a highly ordered and complex crown structure. The printed samples with criss‐crossed layers by interrupting crack propagation exhibit a flexural strength of 134.1 ± 3.9 MPa and a compressive strength of 361.6 ± 8.9 MPa, which are superior to the corresponding values of traditional molding counterparts. The HAp‐monodispersed RBCs are successfully used to print strong and bioactive dental crowns with a printing accuracy of 95%. This new approach can help provide customized components for the clinical restoration of teeth.

## Graphical abstract

Biomimetic dental crown based on hydroxyapatite (HAp)‐monodispersed resin‐based composites (RBCs) is constructed by shear‐induced approach and path control using direct ink writing 3D printing. HAp‐monodispersed RBCs inks can be printed smoothly with programmable high orientation through a new type of custom‐built nozzle. Printed high‐precision crowns with multi‐scale highly ordered structure exhibit outstanding mechanical properties and excellent bioactivity for dental restoration.

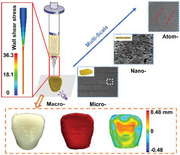

## 1 Introduction

Enamel is a highly calcified hard tissue primarily composed of a dense and orderly arrangement of hydroxyapatite (HAp) nanocrystals.^[^ ^1^ ^]^ It has a high degree of *c*‐axis preferred orientation,^[^ ^2^ ^]^ so as to meet its required properties such as mechanical strength and toughness. The remarkable strength and toughness of these structural materials are conferred through the hierarchical assembly of multi‐scale (i.e., atomic to macro) architecture and components.^[^ ^3^ ^]^

Researchers have been developing bioinspired methods to mimic the unique structure of the dental enamel, such as the biomineralized approach^[^ ^4^ ^]^ and inorganic template synthesis.^[^ ^5^ ^]^ Shao et al.^[^ ^4c^ ^]^ designed a rational material composed of calcium phosphate ion clusters that can be used to produce a precursor layer and induce the epitaxial crystal growth of enamel apatite, by mimicking the biomineralization crystalline‐amorphous frontier of hard tissue development in nature. Yu et al.^[^ ^6^ ^]^ reported a bottom‐up step‐by‐step assembly strategy to build tooth enamel‐mimetic structural materials based on highly ordered ultralong HAp nanowires. Some researchers successfully imitated the distinct multi‐scale aligned structure of natural enamel. However, the above methods can only achieve an orderly arrangement of a single horizontal plane at the nanoscale (10–500 nm), microscale (10–500 µm), or rough macroscopic shape control. Usually, in addition to the outer enamel layers arranged in parallel, natural enamel also has the inner enamel layer with a certain deflection angle.^[^ ^7^ ^]^ More importantly, the natural enamel has a clear and precise macroscopic shape with more than 1 mm in thickness, and 1 cm in size, which further increases the difficulty of preparing biomimetic materials.^[^ ^8^ ^]^

Synthetic HAp has similar physical and chemical properties as the HAp in tooth enamel, which has excellent biocompatibility, structural stability, and abrasion resistance.^[^ ^9^ ^]^ It is widely used as an enhancement material for human tissue repair. Unfortunately, dental composites using HAp particles usually possess lower mechanical properties (flexural strength 40–80 MPa).^[^ ^10^ ^]^ Hydroxyapatite whiskers or nanofibers can possess improved mechanical properties,^[^ ^11^ ^]^ while higher filler loading tends to result in aggregations that serve as mechanical defects. The aggregation influences the filler dispersion in the composite, leading to decrease in the mechanical properties of the dental resin composite.^[^ ^12^ ^]^ Recently, our group successfully prepared monodisperse HAp nanorods with a controllable aspect ratio through a high‐gravity reactive precipitation method in a rotating packed bed (RPB) combined with hydrothermal treatment.^[^ ^13^ ^]^ The resulting HAp nanorods can be used as an excellent filling material to prepare a printable dental restoration composite resin paste.

Direct ink writing (DIW) 3D printing technology based on extrusion can be used for the programmable assembly of 3D architectures.^[^ ^14^ ^]^ Controlled particle orientation is typically achieved by shear‐induced arrangement in the printing process, including alumina platelets and nanowires,^[^ ^15^ ^]^ carbon fibers,^[^ ^16^ ^]^ silicon carbide whiskers,^[^ ^17^ ^]^ and cellulose nanocrystals.^[^ ^18^ ^]^ However, it is challenging to develop viscoelastic inks that can be easily extruded, and yet form a self‐supporting feature after exiting the nozzle. A viscoelastic response with a finite yield stress is required for filament patterning.^[^ ^19^ ^]^

Herein, inspired by the multistage growth of dental enamel, a bottom‐up step‐by‐step strategy was developed using extrusion‐based 3D printing technology to achieve a high‐precision dental crown with multi‐scale (from atom‐ to nano‐ to micro‐ to macro‐scale) highly ordered HAp structure. First, highly dispersible and stable hybrid resin‐based composites (RBCs) with “supergravity +” HAp nanorods were prepared. In this case, “supergravity +” is a two‐step method with supergravity preparation technology and hydrothermal treatment. A high gravity environment (tens to hundreds of *g*) is helpful to achieve the precise controllability of the particle size and distribution.^[^ ^13^ ^]^ The printing nozzle was custom‐built using computational fluid dynamics (CFD) simulation and analysis based on the rheological properties of the slurry, which was conducive to smooth extrusion and stable shearing. The arrangement of HAp was manipulated by adjusting the direction of the printed fibers, and a highly‐ordered and complex crown structure was finally 3D printed using the HAp‐monodispersed RBCs. The HAp‐based RBCs exhibited remarkable mechanical strength, even at low content of HAp, which was favorable for constructing 3D structures with aligned nanofillers. To the best of our knowledge, a highly precise crown over 1 cm with multi‐scale ordered architecture has not yet been 3D printed based on HAp nanostructures. This work opens a door for the development of biomimetic materials with unique structures and functions.

## 2 Results and Discussion

### 2.1 Fabrication and Characterization of HAp‐Based RBCs

Fiber‐shaped HAp is expected to have better mechanical properties compared with HAp particles. The morphology, size, and structure of HAp nanorods are shown in Figure S1 (Supporting Information). The HAp nanorods with average width of 74 nm showed a high aspect ratio up to 35 with average of 21. The energy‐dispersive X‐ray spectrometer (EDS) analysis, high‐resolution transmission electron microscopy (TEM), and X‐ray diffraction (XRD) pattern indicated that the as‐prepared product was pure HAp. The bands at 1724 cm^–1^ (C═O), 1637 cm^–1^ (C═C), and 2960 cm^–1^ (C—H) in the Fourier transform infrared (FTIR) spectrum confirmed the successful modification of *γ*‐MPS with HAp.

**Figure 1:**
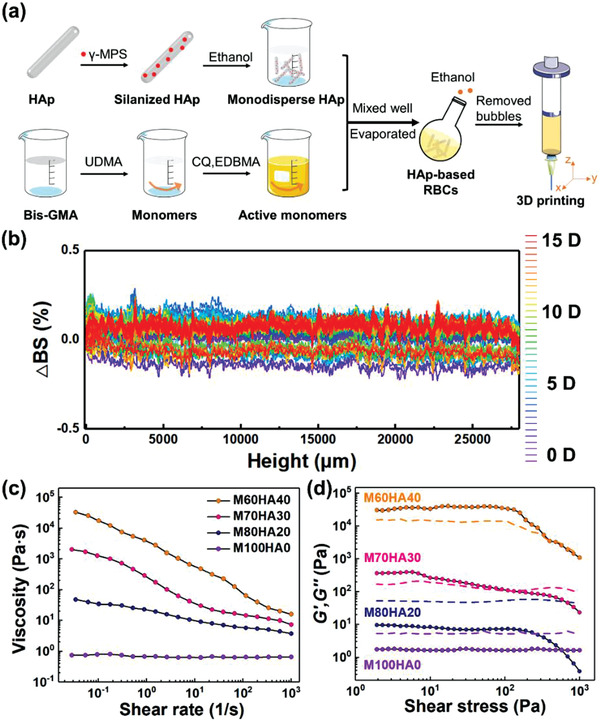
Stability and rheology of HAp‐monodispersed RBCs inks. a) Schematic illustration of preparation process for HAp‐monodispersed RBCs inks. b) Referenced backscattering coefficient (BS) history of HAp‐monodispersed RBCs inks from 0 to 15 d, illustrating the stability of the inks. The inks were scanned in a transparent glass bottle from bottom to top. The first BS profile was subtracted from all other profiles to obtain the backscattering variation (Δ*BS*/%). Rheological data obtained under c) steady‐shear and d) oscillatory conditions for HAp‐monodispersed RBCs inks with M100HA0, M80HA20, M70HA30, and M60HA40. The solid and dashed lines represent storage modulus *G*′ and loss modulus *G*″, respectively.

To solve the problem of agglomeration, the monodisperse HAp nanorods in ethanol were added to active monomers (**Figure** **1**a), instead of the traditional direct mechanical blending of powder. Different from the previous method of preparing RBCs,^[^ ^20^ ^]^ this approach allows HAp to maintain its original nano‐shape evenly dispersed in the organic monomer, thereby further inducing its orientation. Figure S2 (Supporting Information) shows optical microscopy images and rheological data of HAp‐monodispersed inks derived using different methods. Light transmission experiment was performed to characterize the stability and dispersion of the HAp‐monodispersed RBCs inks. The variation of backscattering (△*BS*/%) was around ±0.2% over 15 d of aging at 25 °C, which indicated that the agglomeration and sedimentation of particles were successfully overcome, as shown in Figure 1b. The inks can be stored at 4 °C in the dark for more than one year. Figure S3 (Supporting Information) also shows the photograph of the HAp‐monodispersed RBCs inks after being stored for 13 months at 4 °C.

The effect of HAp nanorods on the RBCs’ rheological behavior was studied through steady‐state and oscillatory measurements. Figure 1c,d shows the steady‐state viscosity and the yield stress (*τ*_y_, the stress at the crossover points of storage modulus *G*′ and loss modulus *G*″) of the inks at 25 °C. In the absence of HAp, the pure resin M100HA0 possessed a viscosity (*η*) of about 0.7 Pa s, exhibiting a Newtonian response (Figure 1c). Besides the lower *G*′ compared to *G*″, both moduli of M100HA0 were independent of the applied shear stress (Figure 1d). Consequently, the resin with low viscosity could not support itself after printing. The addition of HAp nanorods transformed the resin into a viscoelastic fluid with well‐defined apparent yield stress. The viscosity of all the inks clearly showed shear‐thinning behavior, but only the viscosities of M70HA30 and M60HA40 were in the printable range of 10^3^ to 10^5^ Pa s at low shear rates (about 0.01 s^–1^), according to relevant studies.^[^ ^19^, ^21^ ^]^ The lower viscosity under high shear rates resulted in easy flow of the resin through fine nozzles during printing, while the high viscosity under low shear rates allowed the shape retention of the resin after extrusion. With increase in content of HAp nanorods, the yield stress and viscosity under low shear rates of the resin increased and prevented distortion of the printed objects. This indicated that the paste required more shear force to be dispensed, but it was easier to shape after extrusion. The *G*′ plateau over *G*″ indicated solid‐like behavior of the paste at shear stress below *τ*_y_. When the stress was greater than *τ*_y_, the precursor behaved more like a viscous liquid as *G*″ exceeded *G*′. Although M60HA40 could be used as ink for 3D printing, its high viscosity and shear yield stress could lead to remarkably high printing pressures. When the maximum shear stress generated within the nozzle is not high enough to overcome the shear yield stress, a plug flow region develops, leading to an unyielded region of ink whose velocity remains constant. Under these conditions, HAp nanorods cannot be expected to align.

The radial shear stress within the nozzle (*τ*) during direct writing was estimated by the following equation^[^ ^18^, ^22^ ^]^

(1) $$\tau =\frac{\Delta P}{2L}\;\times r$$

where Δ*P* is the maximum pressure applied at the nozzle, *r* is the radial position from the center to the edge of the nozzle, and *L* is the nozzle length. Substituting for the parameters of the 3D printer used in this study (Δ*P*_max_ = 6 × 10^5^ Pa; *r*_max_ = 255 × 10^−6^ m; and *L* = 3.0 × 10^−2^ m), the calculated maximum shear stress (*τ*_max_) at the wall of the nozzle is 2550 Pa. Comparing the dynamic yield stresses of the as‐prepared HAp‐monodispersed RBCs inks with the *τ*_max_ developed in the nozzle, it was found that only inks containing the highest content of HAp nanorods (40 wt%) experienced plug flow (i.e., *τ*_y_ > *τ*_max_). For M70HA30 inks with a *τ*_y_ of 102 Pa, the critical radius (*r*_c_) above which the ink can undergo shear was 10.2 µm for Δ*P* = 6 × 10^5^ Pa. Thus, it is expected that M70HA30 would experience shear forces during extrusion, which can lead to the desired filler alignment. Hence, M70HA30 ink was used to print 3D orientational architectures.

### 2.2 Simulation and Analysis of Custom‐Built Nozzles (CBN) Based on HAp‐Monodispersed RBCs Inks

**Figure 2:**
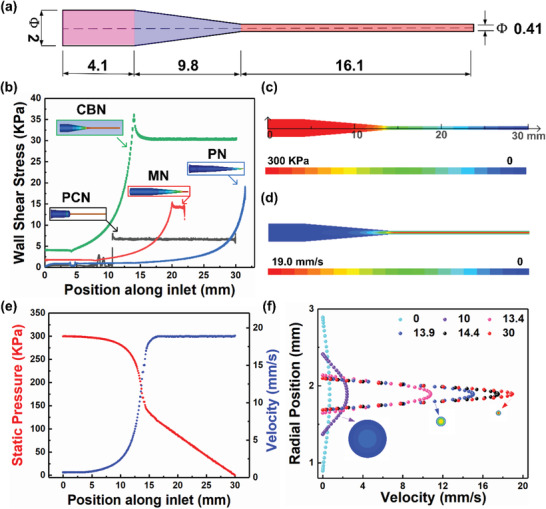
FEM results of CBN based on HAp‐monodispersed RBCs inks along the nozzle channel. a) Schematic showing dimensions of CBN. b) Wall shear stress of four nozzle types along the microchannel. c) Static pressure vector and d) magnitude of the velocity from FEM along the nozzles of 410 µm using the inlet pressure of 300 kPa. e) Cross‐sectionally averaged static pressure (red) and velocity (blue). f) Radial cross‐section velocity profiles at various positions along the channel lengths (legend indicates positions far from the inlet with a unit of mm), the circular image represents the transverse section of nozzles in (c).

The inner channel shape and size confinement of the printing nozzle are crucial factors for the filler alignment and orientation. To optimize the nozzle design, finite element method (FEM) was used to characterize the effect of three commercial nozzles with an inner diameter of 410 µm: plastic nozzle (PN), metal nozzle (MN), and PTFE‐coated nozzle (PCN). All the nozzles were obtained from Nordson EFD and presented in Figure S3a1–c1 (Supporting Information). The wall shear stress results of the simulated fluid flow in the nozzle from FEM analysis with the same inlet pressure of 300 kPa are shown in Figure S3a2–c2 (Supporting Information). PN and MN could not provide stable shear stress output while PCN generated an abrupt increase in stress due to the non‐streamlined connection. The three commercial nozzles were not conducive to the alignment of HAp nanorods during the shear flow. Hence, to optimize the degree of HAp alignment, the optimal conditions for nozzles were identified, as shown in **Figure** **2**a and Figure S4a (Supporting Information). The nozzle channel is composed of three segments, including ink store segment (the inlet segment, Figure 2a, pink), elongation segment (the streamlined segment, Figure 2a, purple), and shear segment (the straight segment, Figure 2a, orange). The wall stress contour of CBN, the 3D model, and digital photograph of nozzle according to the internal channel are shown in Figure S4b,c (Supporting Information).

The CBN not only maintained the streamlined internal structure that was conducive to smooth extrusion, but also increased the outlet section for stable output of shearing force. The wall shear stress gradually increased and maintained a long‐distance high stability along the nozzle outlet (Figure 2b). Figure 2c shows the pressure profile along the nozzle from FEM analysis, using the same inlet pressure of 300 kPa. The velocity profile along the nozzle was also simulated, as shown in Figure 2d. The color map indicates the magnitude of the pressure vector. Figure 2e (red line) shows the detailed static pressure at different positions along the nozzle. The pressure remained at 300 kPa at the ink store segment and then decreased dramatically at the elongation segment and shear segment to 0 MPa. As a result, the large pressure drop provided enough force to increase the velocity and shear force of the HAp‐monodispersed inks. Detailed average velocity data are also shown in Figure 2e (blue line). The velocity increased at a low rate and then rose dramatically to 18 mm s^−1^ with axial distance along the channel. In the shear segment, the velocity remained at almost the same value. The radial cross‐section velocity profiles at various positions along the nozzle lengths (0, 10, 13.4, 13.9, 14.4, and 30 mm far from the inlet) are shown in Figure 2f. It was found that the velocity showed a significant increasing trend along the channel. Moreover, velocity at the central axis was the highest and the radial velocity was much smaller than the axial velocity. In addition, the velocity at the symmetric position along the radial axial was the same. As described above, it is meaningful to focus on the inner channel shape and size confinement of the printing nozzles through the simulation for further 3D printing and particle alignment. According to the above simulation analysis, the internal structure of CBN with different diameters should ensure the same streamline and shear segment. The dimensions of CBN with inner diameters of 260, 330, 410, and 510 µm are presented in Table S1 (Supporting Information).

### 2.3 Shear‐Induced Orientation of HAp‐Based RBCs

**Figure 3:**
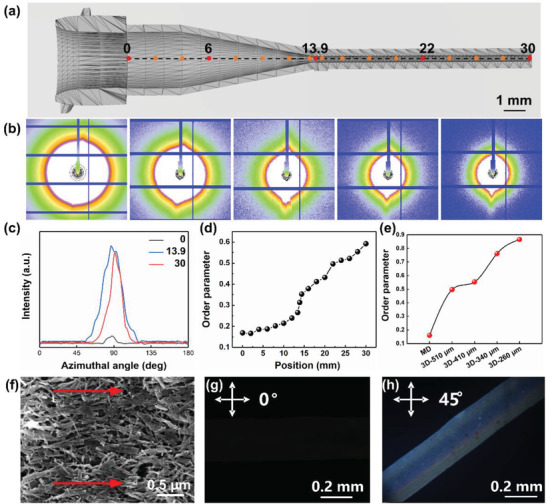
Alignment of HAp‐based RBCs in nozzle. a) Schematic of the nozzle channel geometry used for the SAXS experiments. Circles show the location where in situ measurements were carried out. The diameter of nozzle is 410 µm. b) SR‐SAXS patterns at selected locations (0, 6, 13.9, 22, and 30 mm far from the inlet) in the channel of M70HA30 and c) corresponding azimuthal scan. d) Local order parameters of M70HA30 calculated from SAXS patterns as a function of position in the channel, which is consistent with the white circles shown in (a). e) Order parameters calculated from SAXS patterns for the samples prepared by molding method and 3D printing with nozzles of different diameters. f) SEM images show the HAp‐based RBCs consist of highly ordered HAp nanorods. g,h) POM images of a printed HAp microfiber of HAp‐based RBCs.

It has been reported that aligned structures could be obtained with the assistance of shear force from the nozzle.^[^ ^15^, ^17^, ^18^ ^]^ To evaluate the alignment effect, the small‐angle X‐ray scattering (SAXS) results of the HAp‐monodispersed RBCs inks squeezed through the nozzle channel were characterized, as shown in **Figure** **3**. Schematic of the nozzle geometry used for the SAXS experiments is shown in Figure 3a. Measurements were carried out every 1 mm along the channel, and the measurement locations are shown by the colored circles. To study the change of the junction, test points were set 500 µm before and after the junction of the elongation and shear segments. The initial 2D synchrotron radiation (SR)‐SAXS patterns of the chosen positions are shown in Figure 3b. The 2D patterns showed obvious differences at different positions of nozzles. The higher order of HAp alignment is evidenced by the higher anisotropy of the SAXS streak pattern and the narrower distribution of the intensity in the azimuthal direction (Figure 3c). The order parameter can be obtained by integration according to Equation (4) and is shown in Figure 3d at each position along the nozzle channel. It was found that the order parameter increased from 0.17 to 0.59 along the channel outlet. In a static ink, HAp nanorods are self‐assembled into randomly oriented domains. When HAp‐monodispersed RBCs inks were pumped through the nozzle, HAp tended to be realigned along the extrusion direction by the shear and extension stress from the channel wall.

The order parameters of samples prepared by molding method (MD) and 3D printing with nozzles of different diameters were compared in Figure 3e. The gradual increase in shear stress resulted in the increase in order parameter from 0.50 to 0.86 when the nozzle diameter decreased from 510 to 260 µm compared with the MD (0.16). Scanning electron microscope (SEM) image of HAp nanorods exhibited a highly ordered alignment along the extrusion direction (Figure 3f). The degree of order can be well controlled by adjusting the diameter of the syringe nozzle (Figure S5, Supporting Information). The HAp nanorods arranged randomly with the minimum of free energy in the samples prepared by MD. The disordered HAp nanorods gradually oriented along the printing direction driven by the shear force generated by the nozzle wall during printing extrusion. The order degree further improved from 56% to 97% by decreasing the diameter of nozzle, which was consistent with the SAXS results. In addition, by observing the printed HAp microfiber using the polarizing optical microscope (POM), obvious enhanced light scattering phenomenon was found when rotating the microfiber (Figure 3g,h), indicating the anisotropy of the HAp microfiber resulting from the uniformly oriented HAp nanorods/bundles.^[^ ^6^, ^18^ ^]^ In brief, these data reveal that HAp nanorods are highly oriented along a programmable printing direction.

### 2.4 Construction of 3D‐Printed Tooth Crowns Consisting of Highly Ordered HAp Nanorods

**Figure 4:**
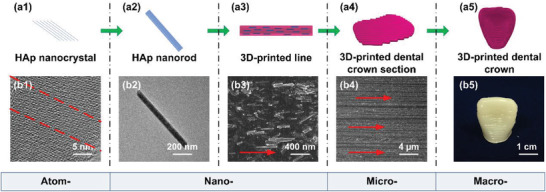
Construction process of the multi‐scale highly aligned HAp nanorod structures. a1–a5) Schematic illustration of the 3D printing process based on the highly ordered HAp‐monodispersed RBCs inks. TEM images of b1) HAp nanocrystal and b2) single HAp nanorod. SEM images of b3) single 3D‐printed line and b4) 3D‐printed dental crown section based on the HAp‐based RBCs. b5) The digital photograph of 3D‐printed dental crown. The red arrows in (b3) and (b4) present the printing direction and printing path, respectively.

In this work, highly aligned multi‐scale (from atom‐ to nano‐ to micro‐ to macro‐scale) HAp nanorod structure was achieved, inspired by the multistage assembly process of the dental enamel, as shown in **Figure** **4**. This work imitated the structure and multistage assembly process of the dental enamel^[^ ^3^, ^5^, ^6^ ^]^ (Figure 4a). Based on the HAp oriented nanocrystalline structure (atomic scale, Figure 4b1), the as‐prepared HAp nanorods (Figure 4b2) corresponded to HAp nanocrystals as the building blocks of the dental enamel. A single highly ordered HAp‐based RBCs fiber (nanoscale, Figure 4b3) was obtained through the shear induction of 3D printing along the printing direction, corresponding to prisms assembled by HAp nanocrystals in the dental enamel. Through the controlled 3D printing path, the HAp‐based RBCs microfiber was formed in parallel (micrometer scale, Figure 4b4), which was similar to the prism orientation for building the macroscopic enamel. A 3D highly ordered HAp‐based RBCs dental crown was further fabricated on a macroscopic scale (Figure 4b5), thereby mimicking the natural structure of the tooth. The highly ordered structure of the as‐prepared complex mainly contributed to its high‐performance functions, thus promoting its biomedical applications.

### 2.5 Mechanical Properties of 3D‐Printed Objects

The tooth nanostructures are oriented almost perpendicular to each other at the dentin–enamel junction in some areas of the natural dental enamel, which can enhance toughness by the mechanism of interrupting crack propagation and forcing cracks to deflect and twist.^[^ ^3^, ^8^, ^23^ ^]^ To mimic the most dominant enhancement mechanisms for high fracture resistance, specimens were designed as mutually vertical layers using the 3D printer software with a print orientation of 0–90° (Figure S6, Supporting Information).

**Figure 5:**
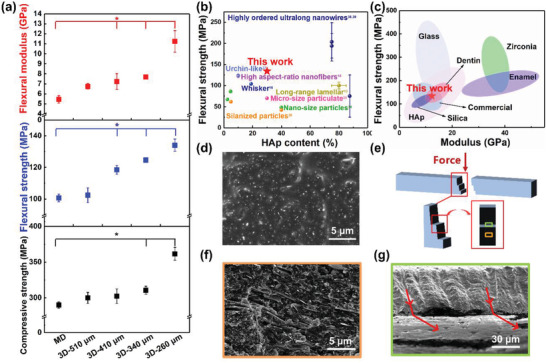
Mechanical properties of 3D‐printed objects. a) Flexural strength, flexural modulus, and compressive strength of the samples prepared by MD and 3D printing with nozzles of different diameters. Data represented as mean ± standard error (*n* = 6), *p*‐values were calculated using one‐way ANOVA. *p < 0.05 means comparison with MD. b) Comparison of the flexural strength‐HAp content of previously reported synthetic HAp‐polymer composites with various structures. c) Comparison of flexural strength modulus of hybrid RBCs reinforced by different ceramic fillers, commercial resin, natural dentin, and enamel.^[^ ^3^, ^7^, ^28^, ^29^, ^32^ ^]^ d) The cross‐sectional SEM images of MD. e) The schematic of the printed cuboids breaking under force. The cross‐sectional SEM images of f) step (orange in (e)) and g) platform (cyan in (e)). The red indicator lines represent the direction of stress deflection. M70HA30 paste was used for all the objects.

Flexural modulus (*E*_Y_), flexural strength (*S*_F_), and compressive strength (*S*_C_) of the HAp‐based RBCs were measured to evaluate the reinforcing effect. The results are presented in **Figure** **5**a. *E*_Y_, *S*_F_, and *S*_C_ increased significantly with decrease in the diameter of nozzles, equivalent to increase in the degree of orientation. In the 3D‐printed M70HA30 with nozzle size of 260 µm, the highest *E*_Y_, *S*_F_, and *S*_C_ values were 11.2 ± 1.1 GPa, 134.1 ± 3.9 MPa, and 361.6 ± 8.9 MPa, respectively, which were higher than those of the molded samples. Figure 5b indicated that the flexural strength of the HAp‐based RBCs was higher than that of many synthetic HAp‐organic composites with various structures such as nano‐size,^[^ ^24^ ^]^ micro‐size,^[^ ^25^ ^]^ and silanized^[^ ^26^ ^]^ particles, whisker,^[^ ^11c^ ^]^ urchin‐like,^[^ ^27^ ^]^ high aspect‐ratio nanofibers,^[^ ^12b^ ^]^ long‐range lamellar,^[^ ^9b^ ^]^ and highly ordered ultralong nanowires.^[^ ^28^ ^]^ Although the strength of some synthetic materials with ordered structure was higher than that of the as‐prepared HAp‐based RBCs, one disadvantage is that higher content of HAp can be hydrolyzed more easily in clinical treatment. The mechanical properties of resin infiltrated ceramic network structure materials with substantial *R* curve behavior,^[^ ^3^, ^29^ ^]^ such as glass and zirconia, were better than those of the simply mixed resin and filler composites, as shown in Figure 5c. It can be clearly seen that the flexural strength of HAp‐based RBCs was equivalent to the commercial resins and matched with the human dentin, which can enable dental replacement with markedly improved performance. The matching of strength is beneficial to avoid the premature failure of either tooth replacement or human tooth as they are at incompatible stress levels.

The mechanism for the improved mechanical properties of the as‐prepared HAp‐based RBCs was further investigated by fracture mechanics analysis. As shown in Figure S6b (Supporting Information), the fracture generated by MD propagated along an approximately straight path and no obvious transversal crack path was observed. However, the crack in the as‐prepared 3D‐printed M70HA30 with the nozzle size of 260 µm propagated along a tortuous path, which is a typical crack deflection phenomenon (Figure S6c, Supporting Information). The SEM (Figure 5d) and microscope images (Figure S6e, Supporting Information) of the MD sample indicate that the fracture surface has a sharp edge. However, the fracture surface of the 3D‐printed sample is rough and has several steps (Figure S6c,f, Supporting Information). The two features were observed in the 3D‐printed SEM fracture section (Figure 5e). First, a zig‐zag microcrack appeared, following the fracture of the HAp nanorods/bundles (Figure 5f). The uneven fracture of HAp nanorods/bundles consumed significant energy, which can enhance the mechanical properties. Second, a stepped section morphology was clearly observed, indicating that the perpendicular interfaces forced the cracks to deflect, which could not reach the adjacent layer (Figure 5g). The highly ordered aligned structure of HAp nanorods can effectively control the crack propagating orientation. In addition, the bonding between HAp nanorods and resin can prevent the crack propagation. It is obvious that the inorganic/organic nanocomposite and hierarchical highly aligned structure synergistically strengthen the RBCs. Therefore, these factors synergistically improved the mechanical properties of the HAp‐based RBCs, similar to the extrinsic strength mechanism in natural complex hierarchical structures.^[^ ^30^ ^]^ The flexural strength after simulated oral environment and Vickers microhardness of the 3D‐printed M70HA30 were higher than or very close to those of the two commercial resins (Esthet‐X and Z350XT) widely used in the market (Figure S7, Supporting Information). The hardness of HAp‐based RBCs (59.4 HV) also matched with the human dentin (58.2–61.2 HV)^[^ ^31^ ^]^ that is helpful to promote the integrity of restoration teeth and the durability of clinical service.

### 2.6 Individual 3D‐Printed Dental Crown

**Figure 6:**
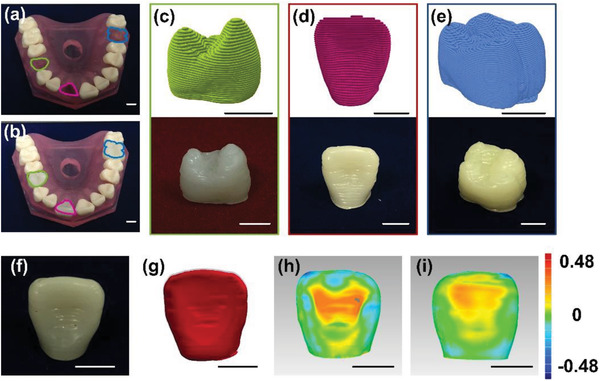
Fine tooth structure fabricated by HAp‐monodispersed RBCs inks. a) Schematic of the three selected crown structures and corresponding opposing dentition. Cyan, rose red, and indigo indicate the crowns of a canine, an incisor, and a molar. b) Fixation of the printed crowns into the model with opposing dentition, with appropriate contact with adjacent teeth and marginal integrity. Sliced file based on 3D model obtained by microcomputed tomography (micro‐CT) from the supporting 3D printing computer software, optical image of the crown structures for the printed c) canine, d) incisor, and e) molar from the top to bottom. f) The photograph and g) 3D model obtained by micro‐CT of polished incisor. Deviation map (mm) of h) front and i) back surface morphologic structure between 3D model of incisor and 3D‐printed polished incisor. The color map indicates the difference between the polished 3D‐printed model and original 3D incisor model where the positive value indicates that the scale of 3D‐printed model is larger than the original one, and vice versa. Scale bar: 1 cm.

Encouraged by the highly ordered HAp‐based RBCs, their customization ability for dental crowns was further demonstrated. To illustrate the structural controllability, molar, incisor, and canine teeth crown structures were prepared as representative examples, as shown in **Figure** **6**a. The inserted molar, incisor, and canine teeth crown structures displayed appropriate contact with adjacent teeth and marginal integrity (Figure 6b). The detailed process of 3D‐printed dental crown preparation is shown in Figure 6c–e. Due to the limited precision during slicing and the needle's diameter in extrusion, the 3D‐printed crown had the lines of the composition. After caries treatment, the restoration material needs to be polished to fit with the natural teeth. The incisor was polished and its 3D model is shown in Figure 6f–i. The polished incisor crown structures exhibited smooth sidewalls and were suitable for dental treatment. Deviation map of front (Figure 6h) and back (Figure 6i) surface morphologic structure showed that the maximum and standard deviation values were ±0.4769 and 0.1392 mm, respectively. The histogram of the deviation is shown in Figure S8 (Supporting Information). The matching accuracy reached 95% based on the crown model. Successful 3D printing of this customized crown indicates that the as‐prepared material can be used as the composite resin inlay combined with adhesive and fiber to repair caries and large areas of crown defects in clinical practice, thus increasing the retention capacity of the tooth.

### 2.7 In Vitro Bioactivity of 3D‐Printed HAp‐Based RBCs

**Figure 7:**
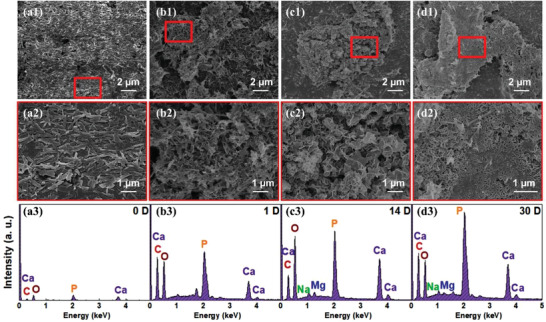
In vitro bioactivity of 3D‐printed HAp‐based RBCs. a1–d2) FESEM images and a3–d3) EDS profiles of the surface of M70HA30 stored in SBF for a) 0, b) 1, c) 14, and d) 30 d.

In vitro bioactivity is defined as the ability of a material to generate a surface apatite layer when submerged in modified‐simulated body fluid (SBF).^[^ ^33^ ^]^ For the dental restorative RBCs, the formed apatite layer could decrease the chances of bacterial aggregation and secondary caries. **Figure** **7** illustrates the changes in surface morphology and chemical composition of the studied HAp‐based RBCs after storage in SBF for different durations. Compared with the specimen before immersion (Figure 7a), dispersed apatite particles were observed on the surfaces of 3D‐printed HAp‐based RBCs after 1 d (Figure 7b). After soaking for 14 and 30 d, the RBCs were covered with a dense and thick layer (Figure 7c,d), which showed a typical platelet morphology with close and continuous stacking. Results of EDS analysis showed that the main elements were carbon, oxygen, calcium, and phosphorus (Figure 7a3–d3). Carbon and oxygen were likely from the resin matrix. For the HAp‐based RBCs before immersion (Figure 7a3), small amounts of calcium and phosphorus were detected, which can be ascribed to the nanorods near the detection point. Dot mappings of the formed particles showed significant amounts of calcium and phosphorus after immersion, which could be from the precipitated apatite layer. The Ca/P atomic ratio ranged from 1.60 to 1.64. The values were less than the stoichiometric ratio 1.67 of HAp,^[^ ^34^ ^]^ implying that the precipitated layer was calcium‐deficient apatite containing other minor elements such as sodium and magnesium.^[^ ^35^ ^]^ These results proved that the remineralization of HAp‐based RBCs possessed desirable in vitro bioactivity, which can not only improve marginal adaptation between restorations and teeth, but can also delay bacterial accumulation and penetration, halting a potentially recurring caries‐active process.

The lower value of HAp‐based RBCs in Table S3 (Supporting Information) indicates that the HAp‐based RBCs exhibited better chemical stability than commercial Esthet‐X and Z350XT after soaking in the artificial saliva for 24 h. Our previous work demonstrated that silicon‐based RBCs possessed excellent cell proliferation, attachment, and metabolic activity.^[^ ^20a^ ^]^ Although the filler used in the previous work was different from HAp, both inorganic fillers have no effect on the cytotoxicity of RBCs, which is mainly attributed to the release of unbound residual monomers into culture environment. The FTIR data (Figure S9, Supporting Information) showed a double bond conversion (*DC*) value of 68.7 ± 0.7% for M70HA30, which is higher than that of P25M59N16 (a sample code of RBCs in our previous work, 63.6 ± 1.4%) and very close to that of Z350XT (68.3 ± 1.0%).^[^ ^20a^ ^]^ Therefore, M70HA30 may have excellent biocompatibility as silicon‐based RBCs. In general, the stability and bioactivity performance indicate that 3D‐printed M70HA30 has great potentials in dental restoration.

## 3 Conclusion

This study presented a shear‐induced approach for preparing a multi‐scale, highly ordered, and custom‐restored tooth crown with excellent mechanical properties using DIW 3D printing. A new type of nozzle with streamlined internal structure and long shear segment was designed to realize smooth extrusion and stable shearing force, which were conducive for the filler alignment and orientation. The results revealed that HAp nanorods were highly oriented along a programmable printing direction, then arranged parallel to the printed fibers, and finally 3D printed into a highly ordered and complex crown structure. The mutually vertical layer of specimens led to higher flexural strength, well over the minimum requirement of ISO 4049 for the repair resin flexural strength of 80 MPa. It is speculated that the HAp‐based RBCs could also be printed to achieve custom‐restored teeth with outstanding mechanical properties and desirable bioactivity for clinical custom tooth restoration. This paper can provide valuable guidance to construct other multi‐scale ordered structures by 3D printing.

## 4 Experimental Section

### Materials

Analytical reagent grade ammonium water (NH_3_·H_2_O), urea (CO(NH_2_)_2_), calcium nitrate tetrahydrate (Ca (NO_3_)_2_·4H_2_O), ammonium hydrogen phosphate ((NH_4_)_2_HPO_4_), and ethanol (C_2_H_5_OH) were purchased from Sinopharm Chemical Reagent Beijing Co. Ltd. Monomers of 2,2‐bis[4‐(2‐hydroxy‐3‐methacryloyloxy propoxy)phenyl] propane (Bis‐GMA) and triethylene glycol dimethacrylate (TEGDMA) were purchased from Sigma‐Aldrich. Camphorquinone (CQ, 97%), ethyl‐4‐dimethylaminobenzoate (4‐EDMAB, 99%), and 3‐methacryloxypropyltrimethoxysilane (*γ*‐MPS, 99%) were purchased from J&K Scientific. Propylamine and cyclohexane solvents were purchased from Sinopharm Chemical Reagent Co., Ltd. (Shanghai, China). All materials were of analytical grade and used as received without any purification. Dentition model (BZ‐KQ059) was bought from Bai Zhou Science Equipment Co., Ltd. (Shanghai, China). SBF was purchased from Phygene Life Sciences Co., Ltd. (Fuzhou, China).

### Synthesis and Silanization of HAp Nanorods

The HAp nanorods were synthesized by a combination of high‐gravity precipitation method in RPB reactor and hydrothermal treatment according to a previous report.^[^ ^13^ ^]^ Briefly, 2 mL of a mixed solution was prepared according to 1:3 molar ratio of NH_3_·H_2_O and CO(NH_2_)_2_ solutions with the same individual concentration of 10 mol L^−1^, which was immediately added into Ca(NO_3_)_2_ solution (100 mL, 0.2 mol L^−1^). Then, Ca (NO_3_)_2_ solution with a flow rate of 200 mL min^−1^ and (NH_4_)_2_HPO_4_ solution (60 mL, 0.2 mol L^−1^) with a flow rate of 120 mL min^−1^ were pumped into the RPB reactor at 80 °C. The rotating speed of the RPB was set at 2500 rpm and reaction time was about 1 s. The entire HAp precursor collected from the outlet of the RPB was immediately transferred to a Teflon autoclave and heated to 200 °C for 2 h. After hydrothermal crystallization, the slurry was filtered and washed with deionized water for three times, and then vacuum‐dried for 24 h at 75 °C. The RPB was described in detail in a previous work.^[^ ^36^ ^]^ The obtained HAp nanorods were then silanized with *γ*‐MPS based on a previously reported method.^[^ ^37^ ^]^

### Preparation of HAp‐Monodispersed RBCs Inks

The silanized HAp (3 g) was dispersed in 97 g of ethanol and sonicated for 5 min. Bis‐GMA (4.158 g) and TEGDMA (2.772 g) were mixed manually to a uniform state, and 0.07 g of photoinitiators (20 wt% CQ and 80 wt% 4‐EDMAB) were added and stirred for 24 h. The resin matrix was added into HAp‐ethanol dispersion. Ethanol was evaporated by using a rotavapor at 80 °C under pressure of 30 bar, yielding a 30 wt% HAp‐monodispersed RBCs ink which was labeled as M70HA30. The resulting ink was further mixed in a speed mixer (DAC 150.1 FVZ‐K, FlackTek, Inc., Germany) at 3000 rpm for 3 min. All the uncured pastes were placed in a vacuum chamber for 8 h to remove air bubbles and then stored in a refrigerator (4 °C). The HAp‐monodispersed RBCs were labeled as M100HA0, M80HA20, M70HA30, and M60HA40, respectively, representing the mass fraction ratios of monomer (M) and HAp (HA). The preparation process of HAp‐monodispersed RBCs inks is displayed in Figure 1a.

### Specimens Prepared by the Molding Method

Prior to curing, the obtained RBCs mixtures were transferred into silicon rubber molds with a specific shape by a resin carrier. After photopolymerization with an LED light‐curing unit (SLC‐VIII B, 430–490 nm, Hangzhou Sifang Medical Apparatus Co., Ltd., Zhejiang, China) for a total of 120 s (60 s on each side, 1200 mW cm^−2^), all the specimens were covered with glass slides to prevent oxygen‐inhibited layers. Then, the cured specimens were carefully retrieved from the molds for further test.^[^ ^20a^ ^]^

### 3D Printing of HAp‐Monodispersed RBCs Inks

Syringes loaded with HAp‐monodispersed RBCs inks were mounted in the BioScaffolder Printer 4.2 (Gesim, Germany). Inks were driven pneumatically through micronozzles of different inner diameters (260–510 µm). The inks were printed onto a glass slide, and extruded under pressures ranging from 100 to 300 kPa at a speed of 1–10 mm s^–1^. Each layer had parallel printed lines apart from each other with one of the angles of 0° and 90°. UV irradiation of 100 mW/cm^2^ from a UV LED (Omni Cure S1500, Lumen Dynamics) was used to pre‐cure this paste layer for 20 s after it was extruded from the syringe. Prior to irradiation, the nozzle was protected from the light to avoid solidifying the paste inside and consequent clogging in the needle. The printed samples were then exposed to a UV LED curing unit for 50 s mm^−1^ with 1200 mW cm^−2^ intensity to complete the curing process.

In addition to tooth crowns, several CAD (computer‐aided design) specific rectangle (25 × 2 × 2 mm, 30 × 20 × 4 mm) and cylinder (Φ 4 × 6 mm, Φ 6 × 4 mm, Φ 1 × 10 mm) models were used for three‐point bending tests, chemical stability, compressive tests, hardness, and in vitro bioactivity tests, respectively.

The tooth model was imaged and reconstructed through a micro‐CT equipment (InspeXio SMX‐225CT FPD, Shimadzu, Japan). After slicing the source file into layer images, the tooth structure could be 3D printed layer‐by‐layer from the nozzle and preliminarily cured for two layers. Finally, HAp‐monodispersed RBCs inks were completely cured into a single tooth structure.

### Morphology Characterization

The specimens’ surface morphology was observed under a SEM (Hitachi S‐4800) at 10 kV. The anisotropy structure was observed using a POM (Leica DM750P) with a 0°/90° crossed polarizer analyzer.

### Paste Stability and Rheology

The physical stability of HAp‐monodispersed RBCs inks for M70HA30 was analyzed by a multisample stability analyzer (Turbiscan TOWER, Formulation, France). Sample scanning was carried out from the bottom to the top of the inks in a special cuvette holder at 25 °C for 15 d. Rheological measurements were carried out using a HAAKE RS150L rheometer (Thermo Fisher Electron Co., Germany) with a 20 mm plate‐plate diameter (gap = 300 µm). The steady shear viscosity of the pastes was measured at specific shear rates from 0.01 to 1000 s^–1^ at 37 °C. Storage and loss moduli were obtained from stress‐controlled oscillatory measurements performed at 1 Hz at ambient temperature. The applied stress in this analysis was increased stepwise until the values were well above the material's yield stress. The yield stress of the different paste formulations was considered as the crossover point of the storage modulus (*G*′) and loss modulus (*G*″).

### FEM Simulations of Hybrid Paste Flowing in Syringe Needle

Finite element fluid flow simulations were conducted in ANSYS R17.0 (ANSYS, Inc., USA) using a mesh of free tetrahedral elements and boundary conditions of 3‐bar inlet pressure and atmospheric pressure at the outlet. An incompressible, non‐Newtonian fluid model was used to simulate the non‐cross‐linked resin's flow using a power‐law, shear rate‐dependent relationship. The density, constant *n* (the flow behavior index), and constant *K* (the consistency coefficient) were obtained empirically. Hybrid composite inks were assumed to obey the following power law^[^ ^38^ ^]^

(2) $$\tau =K{\dot{\gamma }}^{n}$$

where *τ* is the shear stress, $\dot{\gamma }$ is the shear rate, *K* is the consistency coefficient, and *n* is the flow behavior index. For Newtonian fluids, *n* = 1, while for shear thinning fluids, 0 < *n* < 1. Smaller *n* value represents more shear thinning behavior. Dynamic oscillatory rheological data were fitted to obtain the values of *n* and *K*. Zero‐shear viscosity is also reported to be a product of *n* and *K*. Rheology fitting data are shown in Figure S10 and Table S2 (Supporting Information). All the simulations were grid‐independent, and convergence criterion independence after mesh settings was studied. All the discretization errors converged to zero.

### Synchrotron Radiation Small‐Angle X‐Ray Scattering (SR‐SAXS)

SR‐SAXS was carried out with a beamline BL19U2 at the Shanghai Synchrotron Radiation Facility to investigate the shear effect of the printing nozzles. The beam spot size was 50 × 450 µm^2^, the chosen X‐ray wavelength (*λ*) was 0.104 nm, and the corresponding energy was 12 keV, while the sample‐to‐detector distance was 5730 mm with the exposure time of 30 s. As regular stainless‐steel nozzle has obvious X‐ray absorption, a nozzle was prepared with pure resin using a light‐curing 3D printer (Form 2, Formlabs, USA) as background correction. The HAp‐monodispersed inks were scanned for SAXS characterization along the flow direction in the microchannel at a step size of 2 mm. The nozzle device was scanned through a micro‐focused X‐ray beam. The SAXS signal in each position was recorded in the detector plane. Additionally, an optical microscope was placed at the beamline, which can be moved down to facilitate the positioning of the device with respect to the beam. After the azimuthal scans of the lattice plane of these positions were integrated, the alignment was quantified by converting the crystal orientation distributions to the order parameter (*S*) which was calculated according to the following equations^[^ ^39^ ^]^

(3) $$S=\frac{3}{2}\cos^{2}\varphi -\frac{1}{2}$$

where *φ* is the azimuthal angle in a diffractogram. Expanding the average gives

(4) $$S=\int_{0}^{\pi }I\left(\varphi \right)\left\{\frac{3}{2}\cos^{2}\varphi -\frac{1}{2}\right\}\sin\varphi d\varphi$$

which is normalized according to

(5) $$\int_{0}^{\pi }I\left(\varphi \right)\sin\left(\varphi \right)=1$$

where *I*(*φ*) is the intensity distribution along a constant *q*‐value. Order parameter of 1 represents fully aligned fibrils along the microchannel direction, whereas 0 represents an isotropic distribution (random orientation).

### Mechanical Properties

Flexural strength, flexural modulus, and compressive strength were measured using a universal testing machine (Instron 5900, USA), according to literature procedure.^[^ ^40^ ^]^ The dimensions and test parameters of specimens were adjusted according to ANSI/ADA Specification No. 27‐2009 (ISO 4049‐2009). Rectangular and cylindrical specimens were prepared for the three‐point bending test (a span distance of 20 mm, a cross‐head speed of 0.75 mm min^−1^, *n* = 6), and the compressive test (a loading rate of 1 mm min^−1^, *n* = 6), respectively. The specimens were then polished using 2500‐grit sandpaper before the test. The fracture surface morphology of specimens after a three‐point bending test was observed under SEM at 10 kV. The samples were sputter‐coated with gold before observation. The macroscopic fracture surface of specimens was characterized under a digital microscope (Leica DVM6) after a three‐point bending test.

### Construction of 3D‐Printed Tooth Crown Model

The printed crown was manually polished with an electric grinder, using abrasive paste. The polished crown was scanned again by micro‐CT. The 3D graphic consistency between the polished models and real tooth models was compared as the judgment standard.^[^ ^41^ ^]^ Geomagic Qualify 2013 software (Raindrop Geomagic Company, USA) was used to pairwise best‐fit registration between the printed polished 3D crown model and the original one. The magnitude of the difference can be evaluated in the distribution color map.

### In Vitro Bioactivity

The apatite forming ability of the HAp‐based RBCs was evaluated in SBF using a similar method as previously reported.^[^ ^42^ ^]^ Briefly, disc specimens of Φ10 mm × 1 mm were immersed in 20 mL SBF at 37 °C for 1, 14, and 30 d, and the SBF was renewed once a week. Samples were gently rinsed with deionized water and placed in an oven at 60 °C for 6 h before test. The surface morphological change and chemical composition change were measured by SEM equipped with an EDS (Quantax 400, Bruker, Germany).

### Statistics Analysis

For paste stability test, referenced backscattering coefficient was obtained by subtracting the background of the HAp‐monodispersed RBCs inks in the cuvette holder. Data of mechanical properties, Vickers microhardness, and degree of conversion were expressed as mean ± standard deviation of three or more independent measurements. Quantitative analysis was analyzed using Statistical Product and Service Solution 25.0 (SPSS 25.0) software. One‐way ANOVA testing was carried out across groups. In all cases, significance was defined as the probability level *p* ≤ 0.05.

## Conflict of Interest

The authors declare no conflict of interest.

## Supporting information

Supporting Information

## Data Availability

The data that support the findings of this study are available in the Supporting Information of this article.
