# Supplementary material for: 3D‐Printed Strong Dental Crown with Multi‐Scale Ordered Architecture, High‐Precision, and Bioactivity
Source: Adv Sci (Weinh). 2021 Dec 22;9(5):2104001. doi: 10.1002/advs.202104001 (PMC8844577; doi:10.1002/advs.202104001)
Supplement: Supplementary file 1 — Supporting Information [file ADVS-9-2104001-s001.pdf]

WILEY-VCH

ADVANCED SCIENCE
Open Access

Supporting Information

for *Adv. Sci.*, DOI: 10.1002/advs.202104001

3D Printed Strong Dental Crown with Multi-Scale Ordered Architecture, High-Precision, and Bioactivity

Menglu Zhao, Danlei Yang, Suna Fan, Xiang Yao, Jiexin Wang, Meifang Zhu and Yaopeng Zhang**

Supporting Information

3D Printed Strong Dental Crown with Multi-Scale Ordered Architecture, High-Precision, and Bioactivity

Menglu Zhao, Danlei Yang, Suna Fan, Xiang Yao, Jiexin Wang, Meifang Zhu and Yaopeng Zhang**

Experimental

Morphology, Phase and Spectroscopy Characterization: Sample morphology was observed under a transmission electron microscope (TEM, JEOL JSM-2100, Japan) operated at an accelerating voltage of 200 kV. The powder sample was dispersed in C_2H_5OH to a concentration of 0.01 mg/mL by sonication for 30 min and then dropped onto a carbon-film-coated copper grid. The particle size and the aspect ratio of HAp nanorods were obtained based on the measurement of over 100 particles from TEM images with image analysis and processing software (Image-Pro Plus 6.0, Media Cybernetics Inc., U.S.). X-ray diffraction pattern of HAp was collected on an X-ray diffractometer (XRD, D2 Phaser, Bruker, Germany) equipped with Cu Kα radiation (λ= 0.154 nm) in the 2θ range 5-80° at a voltage of 30 kV and a current of 10 mA with a step size of 0.01°. Fourier transform infrared (FTIR) spectroscopy was conducted with potassium bromide tableting method in an FTIR spectrometer (Nicolet 8700, Thermo Fisher, USA). Spectra were recorded in the range 4000-400 cm^{-1} and were acquired with a resolution of 4 cm^{-1} and a total of 32 scans per spectrum.

Mechanical properties in simulated oral environment: The mechanical test samples were soaked in artificial saliva (Phygene Life Sciences Co., Ltd, Fuzhou, China) with different pH

values for 24 hours (acidic environment 5.0, neutral 6.8, alkaline 8.0). The socked samples were washed with deionized water and dried for further strength tests.

Hardness: The hardness of the RBCs was characterized by micro-Vickers hardness tester (HXD-1000TMC/LCD, Shanghai Taiming Optical Instrument Co., Ltd., China) based on disc sample (*Φ* 4×6 mm). The indenter load is 50 GF and the load duration is 10 s during the test.

Chemical stability: Chemical stability test is measured by the change in mass per unit area (cuboid: 30×20×4 mm) according to ISO 6872. Before the test, the mass of the sample was accurately measured as m_1, then soaked in artificial saliva with different pH values at 37 □ for 24 hours (acidic environment 5.0, neutral 6.8, alkaline 8.0). The socked samples were washed with deionized water and dried, and accurately measured the mass m_2, and the mass change (*ML,* mg/cm^2) is as follows:

$$ML = \frac{m_1 - m_2}{S}$$

where *S* is the total surface area of the sample.

Degree of conversion: Degree of conversion (*DC*) of RBCs was analysed on a FTIR spectrometer (Nicolet 8700, Bruker Optik GmbH, Ettlingen, Germany), equipped with an attenuated total reflectance crystal (ATR), operating with 64 scans at a resolution of 4 cm^{-1}. FT-IR spectra were recorded for each RBC before and after curing. Then, DC of each RBC was determined from the ratio of absorbance intensities of aliphatic C=C bond (1640 cm^{-1}) against internal standard of aromatic C=C bond (1600 cm^{-1}). Three trials were conducted for each material. The *DC* (%) was then calculated from formula:

$$DC(\%) = (1 - \frac{(A_{1640}/A_{1600})_{cured}}{(A_{1640}/A_{1600})_{uncured}}) \times 100$$

where A_{1637}/A_{1608} is the height ratio of band 1637 cm^{-1} and band 1608 cm^{-1}.

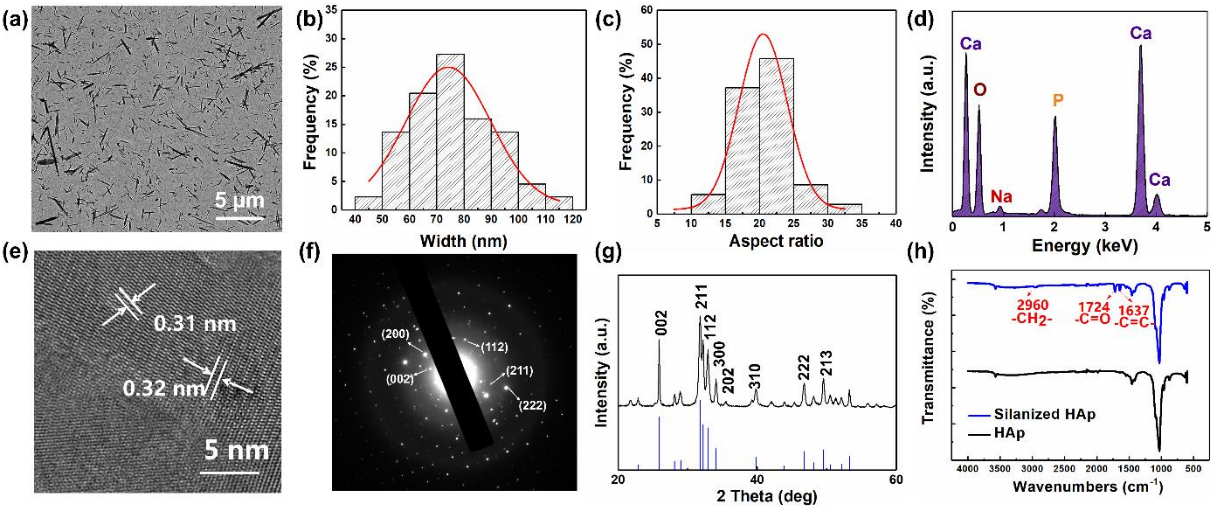

Figure S1. Characterization of morphology, size, and structure of the "supergravity +" HAp nanorods. (a) TEM images, (b) the width and (c) the length distribution of HAp from TEM images. (d) Energy dispersive spectroscopy (EDS), (e) HR-TEM image, (f) a selected-area electron diffraction (SAED) pattern and (g) XRD pattern of the "supergravity +" HAp nanorods. (h) Fourier transform infrared reflection (FTIR) spectra of HAp nanorods and γ-MPS silanized HAp nanorods.

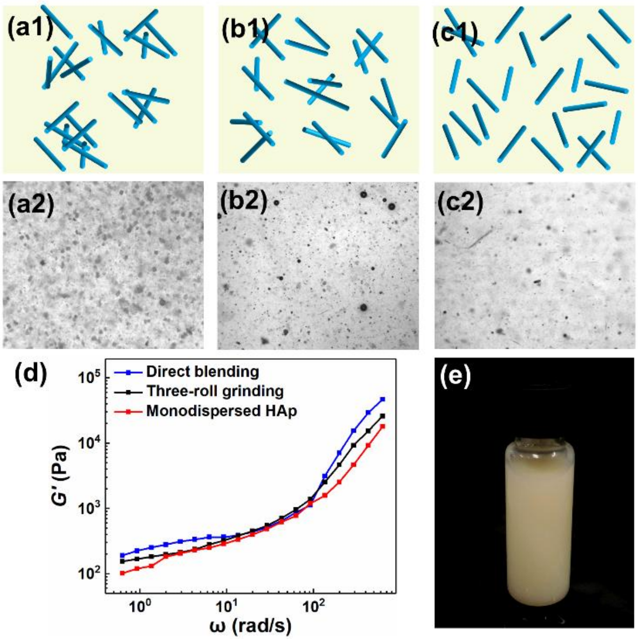

Figure S2. The diagrammatic sketch (a1-c1), optical images (a2-c2) and (d) rheological property of HAp-based RBCs inks using (a1, a2) direct blending, (b1, b2) three-roll grinding

and (c1, c2) monodispersed HAp prepared in this work. M70HA30 was used for all the tests. (e) A photograph of M70HA30 ink after being stored for 13 months at 4 °C in the dark.

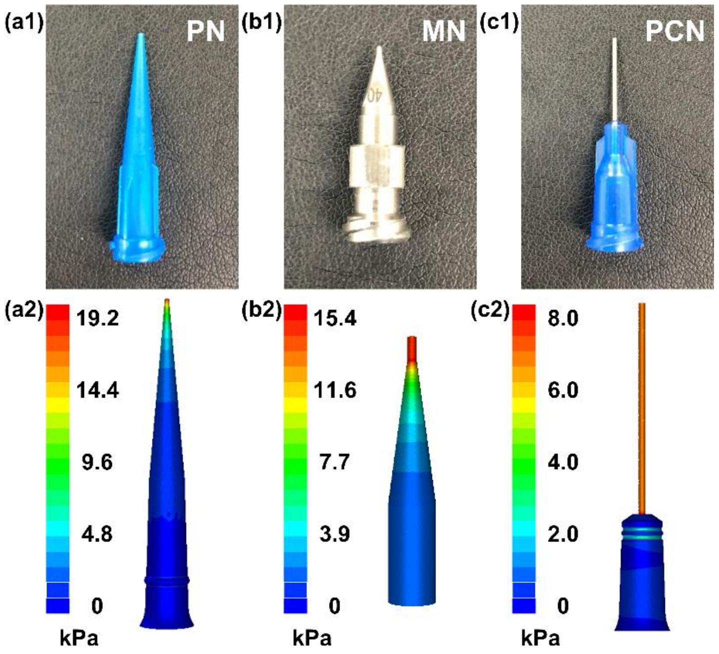

Figure S3. Images of (a1) plastic nozzle (PN, SmoothFlow^{TM} Tapered Tips with polyethylene and polypropylene), (b1) metal nozzle (MN, Precision Nozzles with 303 stainless steel), and (c1) PTFE-coated nozzle (PCN, PTFE-coated tips with stainless steel and PTFE). Detailed data are shown in Table S2, unit: mm. The wall shear stress contours of the simulated fluid flow from FEM analysis in the (a2) PN, (b2) MN and (c2) PCN nozzles with the same inlet pressure of 300 kPa using M70HA30. The diameter of nozzles is 410 μm.

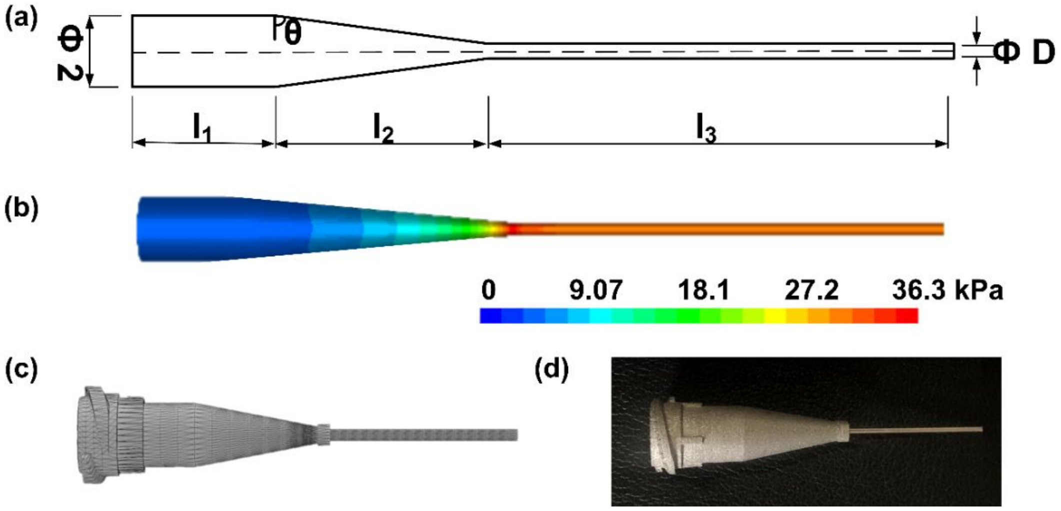

Figure S4. (a) The cross-sectional dimensions of nozzles. Detailed data are shown in Table S2, unit: mm. (b) The wall shear stress contours of the custom-built nozzle (CBN) with a

diameter of 410 µm. (c) 3D model of the CBN according to the internal channel. (d) Digital photograph of the CBN using direct laser sintering 3D printing technology (EOS M290, Germany).

Table S1. Detailed dimensions of print nozzles.

Nozzle-Diameters	l_1 (mm)	l_2 (mm)	l_3 (mm)	Tan θ
CBN-510 µm	4.7	9.2	16.1	12.3
CBN-410 µm	4.1	9.8	16.1	12.3
CBN-330 µm	3.6	10.3	16.1	12.3
CBN-260 µm	3.2	10.7	16.1	12.3
PN-410 µm	11.5	20.0	0	25.2
MN-410 µm	7.2	9.8	2	12.3
PCN-410 µm	10.7	0	19.3	0

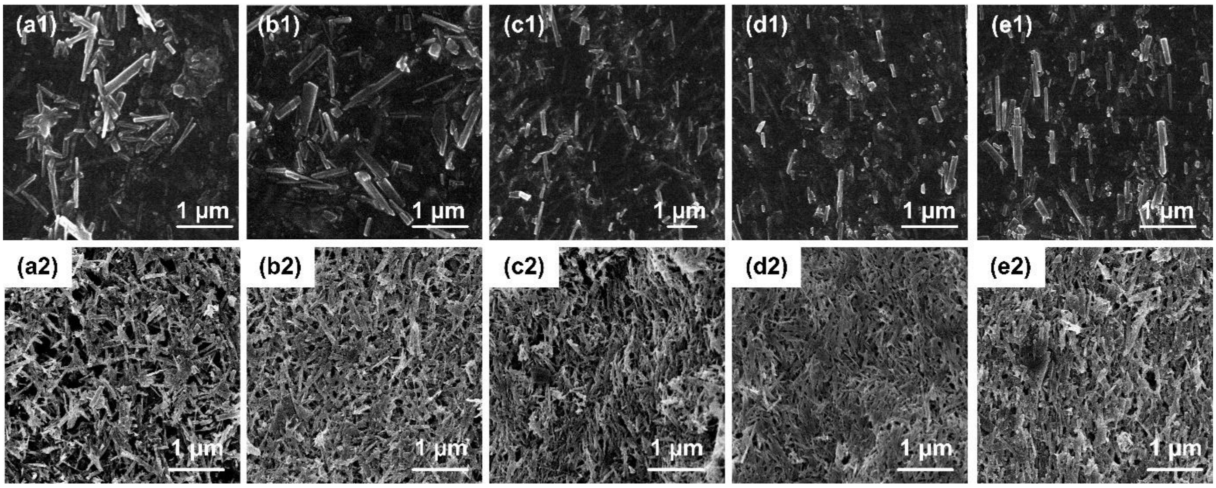

Figure S5. SEM micrographs of the printed samples prepared by (a) MD and 3D printing with nozzle sizes of (b) 510 µm, (c) 410 µm, (d) 330 µm and (e) 260 µm using M70HA30 inks (a1-e1) before and (a2-b2) after alcohol treatment. The degree of order was obtained quantitatively by calculating the ratio of HAp nanorods aligned along the printing direction to the total nanorods based on the representative SEM images.

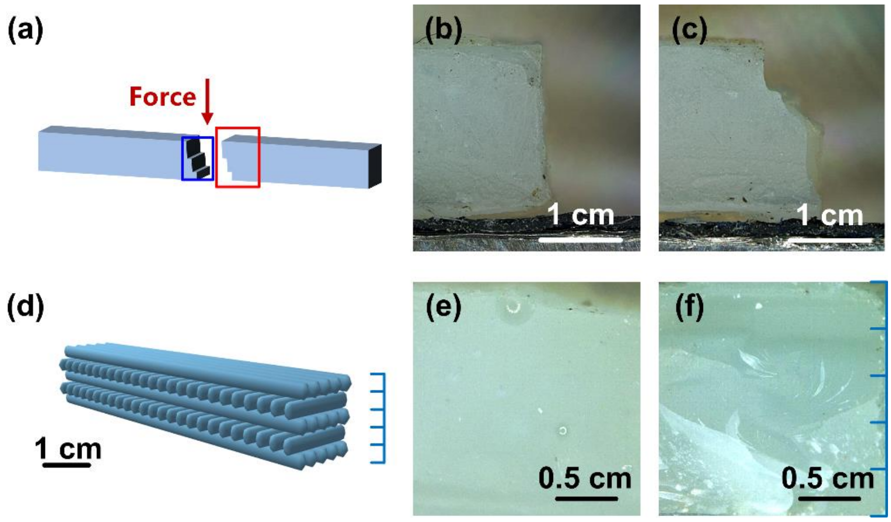

Figure S6. (a) Schematic of the fracture under force. Side view images of specimens prepared by (b) MD and (c) 3D printing after breaking, as indicated in the red box in (a). (d) Print orientation design of distinct internal architectures with angles of 0°/90°. Cross-sectional images of cured cuboids prepared by (e) MD and (f) 3D printing after a three-point bending test, as indicated in the blue box in (a). The light blue line in (f) matches with the layer of 3D printed samples. A Leica DVM6 digital microscope was used to observe the optical images in (b-c) and (e-f).

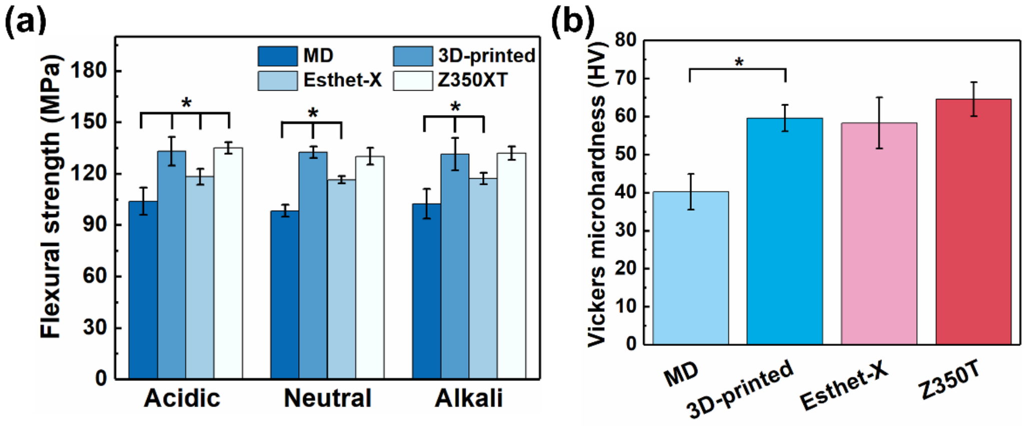

Figure S7. (a) Flexural strength (n=3) after simulated oral environment and (b) Vickers microhardness (n=6) of the objects prepared by MD and 3D printing based on M70HA30, Esthet-X (Dentsply flowable resin) and Z350T (3M ESPE general resin). Data represented as mean ± standard error. *P*-values were calculated using one-way ANOVA. *$p<0.05$ means comparison with 3D-printed.

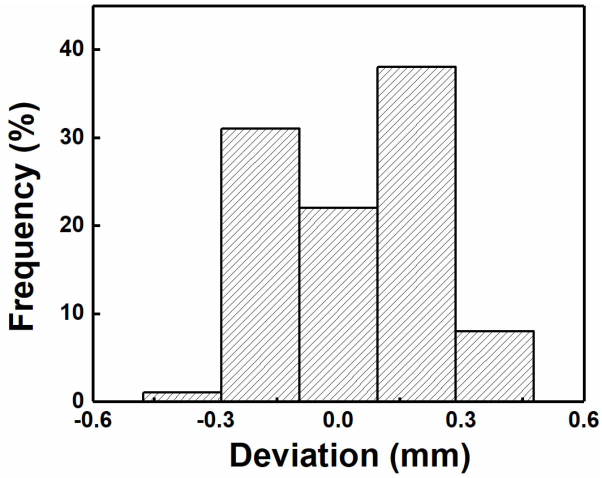

Figure S8. Deviation histogram of surface morphologic structure between 3D model of incisor and 3D printed polished incisor.

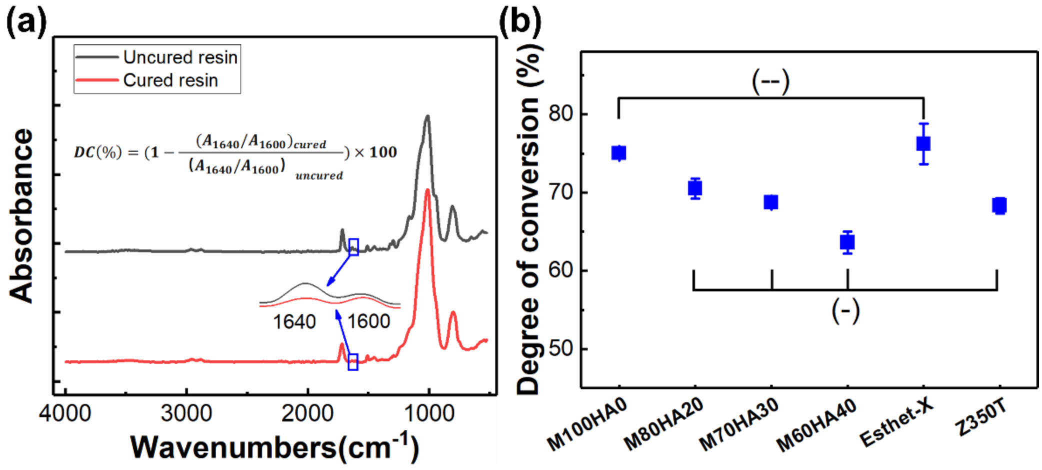

Figure S9. (a) FTIR spectra of uncured and cured M100HA0 resin. (b) Degree of conversion of experimental RBCs reinforced with HAp nanorods of different mass ratios, as well as commercial Esthet-X and Z350XT. Data represented as mean ± standard error (n=3). *P*-values were calculated using one-way ANOVA. (-) $p>0.05$ means comparison with Z350T. (--) $p>0.05$ means comparison with Esthet-X.

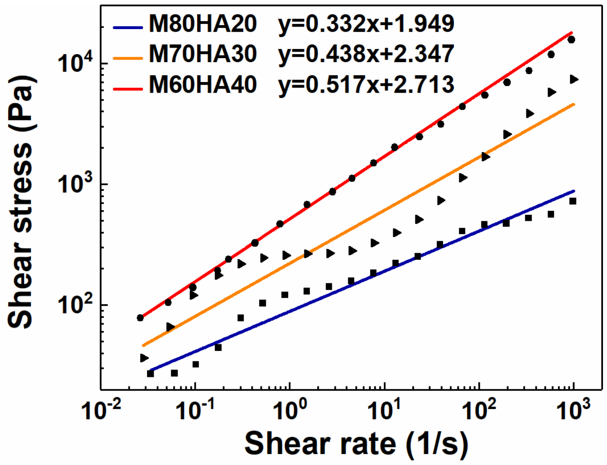

Figure S10. Empirical determination of flow behavior index, n, and consistency coefficient, K, from fitted rheological data for hybrid materials M80HA20, M70HA30 and M60HA40. The black spots represent the original rheological data. Detailed data are shown in Table S1.

Table S2. Flow behavior index n, consistency coefficient K, and zero-shear viscosity η of hybrid composite pastes.

Sample code	n	K [Pa·s^n]	η [Pa·s]
M80HA20	0.332	88.920	29.521
M70HA30	0.438	222.331	97.381
M60HA40	0.517	516.416	266.987

Table S3. The chemical stability (*ML*, mg/cm^2) of the HAp-based RBCs after soaking in the artificial savila for 24 h.

**Samples**	**MD**	**3D-printed**	**Esthet-X**	**Z350XT**
Acidic pH=5.0	0.052	0.018	0.024	0.024
Neutral pH=6.8	0.054	0.034	0.051	0.042
Alkali pH=8.0	0.036	0.034	0.042	0.042
